# Cost-effectiveness of cranial implants compared with autologous bone grafts

**DOI:** 10.1016/j.bas.2025.104217

**Published:** 2025-02-19

**Authors:** Vita M. Klieverik, Pierre A. Robe, Marvick S.M. Muradin, Peter A. Woerdeman

**Affiliations:** a Department of Neurology and Neurosurgery, University Medical Center Utrecht, Utrecht, the Netherlands; b Department of Oral and Maxillofacial Surgery, University Medical Center Utrecht, Utrecht, the Netherlands

**Keywords:** Craniectomy, Cranioplasty, Cost-effectiveness, Autologous bone grafts

## Abstract

**Introduction:**

Autografts are considered more cost-effective than cranial implants due to the use of the patient's own bone. However, autografts are associated with a higher revision surgery rate because of their intrinsic risk of resorption. As revision surgeries imply additional hospital stays and therefore higher costs, autografts may be less cost-effective than cranial implants.

**Research question:**

To analyze the cost-effectiveness of cranial implants compared with autografts.

**Material and methods:**

We performed a retrospective cohort study of patients who underwent cranioplasty between 2014 and 2020. We collected data on the costs of cranioplasty using each patient's diagnosis and treatment combination (DBC) code. We used the incremental cost-effectiveness ratio (ICER) to assess cost-effectiveness, which was calculated as the ratio of incremental cost and incremental effect of using a cranial implant instead of an autograft.

**Results:**

A total of 168 patients were included (mean age 43.0 ± 20.0 years). The median cost of the first cranioplasty procedure was €6249.37 (IQR €5250.64 – €8551.36) for cranial implants and €6261.36 (IQR €5189.14 – €7792.10) for autografts (p = 0.094). The median total cost of all health care related to the cranioplasty procedure was €6460.64 (IQR €6039.68 – €9533.03) for cranial implants and €12,075.01 (IQR €6409.63 – €16,420.71) for autografts (p < 0.001). The ICER of cranial implants compared with autografts was –€7663.22 per revision surgery avoided.

**Discussion and conclusion:**

This study found that the use of cranial implants is at a lower cost and more clinically effective than the use of autologous bone grafts.

## Highlights

• The use of cranial implants has shown to be at a lower cost and more clinically effective than the use of autologous bone flaps.
• The median cost of the first cranioplasty procedure did not differ significantly between cranial implants and autologous bone grafts.
• However, the median total cost of all health care related to the cranioplasty procedure was significantly lower for cranial implants than for autologous bone flaps.
• This suggests that although cranial implants themselves are not significantly cheaper than autologous bone grafts, they are more cost-effective over an extended period of time since they have a lower revision surgery rate and therefore lower total healthcare costs.

## 1 Introduction

Craniectomy is a regularly performed neurosurgical procedure mainly used to decrease medically refractory elevated intracranial pressure (ICP) resulting from traumatic brain injury (TBI), vascular disease, or various other conditions (Brown and Wijdicks, 2017). With advances in medical and surgical care, more craniectomy patients survive their initial insult and eventually require subsequent cranioplasty to protect the underlying dura and brain from physical insult and to restore cosmesis. Cranioplasty also contributes to neurological recovery by reversing abnormalities in cerebral blood flow, cerebrospinal fluid (CSF) hydrodynamics, and cerebral metabolic activity resulting from craniectomy (Fodstad et al., 1984; Winkler et al., 2000).

While often considered a routine surgery, high rates of postoperative complications have been reported after cranioplasty. Bone flap resorption rates range from 4% to 33% in adults and up to 58% in pediatric patients and surgical site infections occur in 2%–24% of patients (Shepetovsky et al., 2021; Klieverik et al., 2019). These complications usually require removal of the cranioplasty implant and insertion of a new one. Recent studies have shown that autologous bone flaps require significantly more revision surgeries than cranial implants, primarily because of the intrinsic risk of bone flap resorption (Malcolm et al., 2018; Klieverik et al., 2023). This finding challenges the prevailing assumption that autologous bone grafts are inherently more cost-effective than cranial implants due to the use of the patient's own bone, since revision surgeries imply additional hospital stays and therefore higher health care costs (Hayward, 1999). This underscores the need to reassess the cost-effectiveness of autologous bone flaps compared with cranial implants. Different studies have evaluated the mean costs of various cranioplasty implant materials, but none have performed a cost-effectiveness analysis to compare the tradeoff between costs and benefits between different cranioplasty implant materials (Binhammer et al., 2020; Baldia et al., 2022; Mrad et al., 2017; Gilardino et al., 2015; Dowlati et al., 2022; Ernst et al., 2018; Lethaus et al., 2014). Therefore, the aim of the present study was to analyze the cost-effectiveness of cranial implants compared with autologous bone grafts.

## 2 Material and methods

### 2.1 Study design and study population

We performed a retrospective cohort study to identify all consecutive patients who underwent cranioplasty following craniectomy from January 1, 2014 to December 31, 2020 at the University Medical Center Utrecht, the Netherlands. Patients were eligible for inclusion if a minimum of one-year follow-up data was available. Patients were excluded if they underwent cranioplasty for the treatment of craniostenosis or craniosynostosis or if they required skull base reconstruction. This study was reviewed and approved by our institution's Medical Research Ethics Committee (MREC) under number 22/159. All patients, except for three, provided informed consent. Sharing of the current data for further research is considered upon request.

### 2.2 Surgical techniques

Patients underwent craniectomy for evacuation of an intracranial mass lesion resulting from TBI or for treatment of otherwise medically refractory raised ICP, or bone flap infection (Brown and Wijdicks, 2017). The time interval between craniectomy and cranioplasty was dependent on the patient's general health status and preference, the treating neurosurgeon's expert opinion, and disponibility of time in the operating room. Prior to the cranioplasty procedure, patients received a prophylactic dose of cephazolin 2000 mg or clindamycin 600 mg. A wound drain was inserted according to the surgeon's preferences. In the case of subsequent autologous cranioplasty, removed bone flaps were preserved under sterile conditions in a freezer at −80 °C or subcutaneously in an abdominal pocket. The surgical incision was closed using absorbable subcutaneous sutures and either skin sutures or staples. When present, the wound drain was removed within the first three days. A clinical follow-up visit was scheduled at six weeks following the cranioplasty procedure and any further follow-up visits were planned individually via the outpatient clinic.

### 2.3 Data collection

We collected data on the costs of cranioplasty by using each included patient's *diagnosis and treatment combination* (DBC) code for any healthcare that was received related to the patient's cranioplasty procedure. DBC codes form the healthcare billing framework in the Netherlands. DBC codes are packages of healthcare products that are required to make a diagnosis and perform the associated treatment (Struijs and Baan, 2011). Using DBC codes, we collected data on the costs in euros of the following provided healthcare related to the patient's cranioplasty procedure.

- The first cranioplasty procedure, including all pre-, per- and postoperative healthcare provided during the hospital stay.
- The standard clinical follow-up visit at six weeks following the cranioplasty procedure.
- Any diagnostic tests (e.g., imaging or laboratory tests) required to help diagnose a possible complication related to the cranioplasty procedure.
- Any revision surgery required because of a postoperative complication related to the cranioplasty procedure (e.g., bone flap resorption or surgical site infection). A revision surgery was defined as any surgery performed to remove the cranioplasty implant and replace it with a new one, either during the same procedure or at a later stage.
- Any subsequent clinical follow-up visits and diagnostic tests required after any revision surgery.

### 2.4 Statistical analysis

Descriptive statistics were used to summarize demographic and outcome variables. Normally distributed continuous variables were presented as means ± standard deviations (SD). Skewed distributed continuous variables were expressed as medians with corresponding interquartile ranges (IQR). Categorical variables were shown as numbers with corresponding percentages.

The primary outcome measure was the cost-effectiveness of cranial implants compared with autologous bone flaps. To assess this outcome measure, we calculated and presented the incremental cost-effectiveness ratio (ICER) (Sanders et al., 2019). The ICER was calculated as the ratio of the incremental cost and the incremental effect of using a cranial implant instead of an autologous bone graft, i.e., by dividing the difference in average cost by the difference in average effect between the cranial implant group and the autologous bone flap group:

$$\text{ICER}=\frac{cost\;\left(cranial\;implants\right)-cost\;\left(autografts\right)}{effectiveness\;\left(cranial\;implants\right)-effectiveness\;\left(autografts\right)}$$

The average cost was defined as the mean total cost of all healthcare related to the cranioplasty procedure, including the first cranioplasty procedure, the standard clinical follow-up visit, and any other required diagnostic tests, revision surgeries, or additional clinical follow-up visits within the follow-up period. The average effect was defined as the proportion of patients without the need for a revision surgery within the follow-up period (i.e., the proportion of revision surgeries avoided). Therefore, the ICER allows for estimation of the additional cost or the cost-saving per revision surgery avoided when using cranial implants over autologous bone grafts.

We performed three sensitivity analyses to explore the uncertainty in the ICER (Sanders et al., 2016; Jain et al., 2011). First, we conducted a ‘worst case’ sensitivity analysis using the maximal total cost instead of the mean total cost to explore parameter uncertainty in the ICER. Since for some patients the total cost of all health care related to the cranioplasty procedure is higher than the mean total cost, the incremental cost in the original ICER may be underestimated and therefore the original ICER may be overly optimistic. Second, a sensitivity analysis using the median total cost instead of the mean total cost was performed to investigate methodological uncertainty in the ICER. Because the total cost of all health care related to the cranioplasty procedure follows a right-skewed distribution, the incremental cost in the original ICER might be overestimated, leading to an unfavorable representation of the ICER. The third sensitivity analysis excluded the subgroup of patients that underwent craniectomy because of an infection, as this subgroup may differ in patient-specific and cranial implant-specific aspects from those patients who underwent craniectomy because of trauma or vascular disease.

The secondary outcome measures included the differences between the cranial implant group and the autologous bone flap group in 1) the expenditure associated with the first cranioplasty procedure, including all pre-, per-, and postoperative health care provided during the hospital stay and 2) the total cost of all healthcare related to the cranioplasty procedure. Because these two outcome measures were both non-normal with skewed-right distributions, we used non-parametric tests to test for differences in median values between the cranial implant group and the autologous bone graft group. P-values <0.05 were taken to indicate statistically significant differences. All statistical analyses were performed using R statistical software, version 4.0.2. (R Foundation for Statistical Computing, Vienna, Austria).

## 3 Results

### 3.1 Baseline characteristics

A total of 182 consecutive patients underwent cranioplasty following craniectomy within the study timeframe. Of this cohort, data on the costs of cranioplasty were available for 168 patients (92.3%). Of these patients, 91 (54.2%) were male and the mean age at cranioplasty was 43.0 ± 20.0 years (range 0.7–83.4 years). The mean cranial defect size was 82.3 ± 32.1 cm^2^ (range 7.9–231.4 cm^2^). The median time interval between craniectomy and cranioplasty was 20.6 weeks (IQR 16.3–29.8 weeks, range 38 days–1.7 years). Autologous bone flaps were used as the cranioplasty implant material in 111 cases (66.1%) and cranial implants were used in 57 cases (33.9%). The baseline characteristics are shown in Table 1. The median follow-up after cranioplasty was 5.1 years (IQR 3.8–6.5 years, range 1.1–37.9 years).

**Table 1:** Baseline characteristics of 168 patients undergoing cranioplasty following decompressive craniectomy (all data given as number of patients (%) unless otherwise indicated).

Characteristic	Value
Males	91 (54.2)
Mean age at craniectomy ± SD (years)	42.4 ± 20.0
Mean age at cranioplasty ± SD (years)	43.0 ± 20.0
Age categories at cranioplasty (years)	
< 18	21 (12.5)
18–50	67 (39.9)
> 50	78 (46.4)
Indication for craniectomy	
Trauma	65 (38.7)
Vascular disease	62 (36.9)
Infection	41 (24.4)
Mean cranial defect size ± SD (cm^2^)	82.0 ± 32.1
Cranial defect size categories (cm^2^)	
< 75	51 (30.4)
≥ 75	108 (64.3)
Median time interval between craniectomy and cranioplasty (IQR, wks)	20.6 (16.3–29.8)
Time interval between craniectomy and cranioplasty categories	
< 12 weeks	12 (7.1)
≥ 12 weeks	154 (91.7)
Cranial reconstruction material	
Autologous bone flap	111 (66.1)
Glass-fiber reinforced composite	35 (20.8)
MMA	14 (8.3)
PMMA	5 (3.0)
PEEK	2 (1.2)
Custom-made porous hydroxyapatite	1 (0.6)
In-hospital complications	6 (3.6)
EDH	3 (1.8)
Wound leakage	2 (1.2)
Epidural hygroma	1 (0.6)
Median follow-up after craniectomy (IQR, yrs)	5.6 (4.3–7.0)
Median follow-up after cranioplasty (IQR, yrs)	5.1 (3.8–6.5)

Missing values were present for age at craniectomy (0.6%), age at cranioplasty (1.2%), cranial defect size (5.4%), time interval between craniectomy and cranioplasty (1.2%), follow-up after craniectomy (0.6%) and follow-up after cranioplasty (1.2%). SD = standard deviation, IQR = interquartile range, MMA = methyl methacrylate, PMMA = polymethyl methacrylate, PEEK = polyetheretherketone, EDH = epidural hematoma.

### 3.2 Cost outcomes

The median cost of the first cranioplasty procedure, including all pre-, per- and postoperative healthcare provided during the hospital stay, was € 6249.37 (IQR € 5250.64 – € 8551.36, range € 2415.42 – € 43,563.71) for the cranial implant group and € 6261.36 (IQR € 5189.14 – € 7792.10, range € 3296.80 – € 39,733.92) for the autologous bone graft group (p = 0.094). The mean cost of the first cranioplasty procedure was € 8782.64 ± € 8502.10 for the cranial implant group and € 7659.79 ± € 4894.32 for the autologous bone graft group. The median total cost of all healthcare related to the cranioplasty procedure, including the first cranioplasty procedure, the standard clinical follow-up visit, and any other required diagnostic tests, revision surgeries or additional clinical follow-up visits, was € 6460.64 (IQR € 6039.68 – € 9,533,03, range € 2566.64 – € 100,620.24) for the cranial implant group and € 12,075.01 (IQR € 6409.63 – € 16,420.71, range € 4051.65 – € 117,470.13) for the autologous bone flap group (p < 0.001). The mean total cost of all healthcare related to the cranioplasty procedure was € 11,861.48 ± € 15,654.60 for the cranial implant group and € 14,168.11 ± € 13,347.31 for the autologous bone graft group, representing a cost-saving of € 2306.63 on average.

### 3.3 Effectiveness outcomes

Out of hospital postoperative complications stratified by cranioplasty implant material are shown in Table 2. The total complication rate was 7 (12.3%) for the cranial implants and 53 (47.7%) for the autologous bone flaps (p < 0.001). A first revision surgery (second cranioplasty implant) was required in 8 cases (14.0%) in the cranial implant group and in 49 cases (44.1%) in the autologous bone graft group (p < 0.001). Consequently, the proportion of patients without the need for a revision surgery (i.e., the proportion of revision surgeries avoided) within the follow-up period was 49/57 (86.0%) in the cranial implant group and 62/111 (55.9%) in the autologous bone flap group. Thus, the use of cranial implants represented on average a 30.1% reduction in the number of first revision surgeries following cranioplasty.

**Table 2:** Out of hospital complications and revision surgeries following cranioplasty (all data given as number of patients (%) unless otherwise indicated).

Event	Autologous bone flaps (n = 111)^b^	Cranial implants (n = 57)^c^	p-value^a^
Total patients with complication	53 (47.7)	7 (12.3)	<0.001
Bone flap resorption	40 (36.0)	0 (0.0)	<0.001
Surgical site infection	15 (13.5)	4 (7.0)	0.208
Mechanical complications	2 (1.8)	1 (1.8)	0.982
Hydrocephalus	1 (0.9)	1 (1.8)	0.629
Implant exposure	1 (0.9)	1 (1.8)	0.629
Pain	0 (0.0)	1 (1.8)	0.162
First revision surgery	49 (44.1)	8 (14.0)	<0.001

This explains the discrepancy between total patients with complication and the stratified number of complications.

a Chi-square test or Fisher's exact test (for cell count less than five) for difference in proportions.

b 5 patients experienced both resorption and infection, and 1 patient experienced both resorption and hydrocephalus.

c 1 patient experienced both infection and hydrocephalus.

### 3.4 Cost-effectiveness

The ICER of cranial implants compared with autologous bone grafts was –€ 7663.22 per revision surgery avoided:

$$\text{ICER}=\frac{€\;11,861.48-€\;14,168.11}{0.860-0.559}=–€\;7,663.22$$

### 3.5 Sensitivity analyses

The ICER of cranial implants compared with autologous bone flaps using the maximal total cost instead of the mean total cost was –€ 55,979.70 per revision surgery avoided:

$$\text{ICER}=\frac{€\;100,620.24-€\;117,470.13}{0.860-0.559}=–€\;55,979.70$$

The ICER using the median total cost instead of the mean total cost was –€ 18,652.39 per revision surgery avoided:

$$\text{ICER}=\frac{€\;6,460.64-€\;12,075.01}{0.860-0.559}=–€\;18,652.39$$

The ICER using the mean total cost of the study population in which the subgroup of patients that underwent craniectomy because of infection were excluded was –€ 5232.33 per revision surgery avoided:

$$\text{ICER}=\frac{€\;13,021.68-€\;14,685.56}{0.844-0.526}=–€\;5,232.33$$

## 4 Discussion

The present study evaluated the cost-effectiveness of cranial implants compared with autologous bone grafts.

We found that the use of cranial implants has shown to be at a lower cost and more clinically effective than the use of autologous bone flaps with an ICER of –€ 7663.22 per revision surgery avoided. The ICER is a single metric that considers the tradeoffs of costs and benefits between alternative interventions and therefore allows for an analytic method for quantifying their cost-effectiveness (Baldia et al., 2022). A negative ICER can result from a negative incremental cost or a negative incremental effect. Our negative ICER results from a negative incremental cost (i.e., using cranial implants results in lower costs compared with using autologous bone grafts) and a positive incremental effect (i.e., using cranial implants results in a higher proportion of patients without the need for a revision surgery compared with using autologous bone flaps). Since cranial implants represented the dominant intervention in our study (i.e., lower cost and more clinically effective), it was unnecessary to source a cost-effectiveness threshold. Therefore, monetary net benefit and health net benefit ratios were not calculated (Baldia et al., 2022; Mrad et al., 2017).

In addition, we found that the median total cost of all health care related to the cranioplasty procedure was significantly lower for cranial implants than for autologous bone grafts (€ 6460.64 versus € 12,075.01, respectively, p < 0.001), while the median cost of the first cranioplasty procedure only did not differ significantly between the two groups (€ 6249.37 versus € 6261.36, respectively, p = 0.094).

The generated ICER's in the sensitivity analyses remained negative when we used the maximal total cost and the median total cost instead of the mean total cost and when we excluded the subgroup of patients who underwent craniectomy because of an infection. This suggests that the use of cranial implants is still at a lower cost and more clinically effective than the use of autologous bone flaps when adjusting for these uncertainties in the ICER.

Our results indicate that although cranial implants themselves are not significantly cheaper than autologous bone flaps and in some individual cases may be even more expensive due to their custom-made design, they are cheaper and more cost-effective over an extended period of time since they have a lower revision surgery rate and therefore lower total healthcare costs. This finding is the main difference between the results from our study and the data currently available in the literature. Previous studies on cost differences between cranioplasty implant materials have reported that the use of cranial implants is more expensive than the use of autologous bone grafts (Binhammer et al., 2020; Gilardino et al., 2015; Dowlati et al., 2022; Ernst et al., 2018; Lethaus et al., 2014). However, these studies reached this conclusion based on the average cost of the first cranioplasty procedure only without considering any additional healthcare costs due to postoperative complications or revision surgeries. Mrad et al. drew a similar conclusion to our study and found that if the price of the cranioplasty implant material itself is not accounted for, cranial implants are less expensive than autologous bone flaps due to less additional health care costs (Mrad et al., 2017). To the best of our knowledge, there was one study by Lethaus et al., also performed in the Netherlands, on cost differences between cranioplasty implant materials that also collected data in euros to which we could compare the average cost of the first cranioplasty procedure (Lethaus et al., 2014). We found that the average cost of both cranial implants and autologous bone grafts was lower in our cohort than in that of Lethaus et al. (€ 8782.64 versus € 15,532.08 for cranial implants and € 7659.79 versus € 10,849.91 for autologous bone grafts) (Lethaus et al., 2014). This may be explained by the fact that their cohort exclusively included titanium and PEEK cranial implants, which may be more expensive than the cranial implants utilized in our study. There were no similar studies available to which we could compare the average total cost of all healthcare related to the cranioplasty procedure.

### 4.1 Strengths and limitations

An important strength of our study is that it is the first to perform a cost-effectiveness analysis to compare the tradeoff between costs and benefits between cranial implants and autologous bone flaps. Instead of only reporting the costs of these different cranioplasty implant materials, we also assessed their effectiveness and combined these outcomes into the single metric of the ICER. The ICER is a reliable analytic method for quantifying cost-effectiveness (Baldia et al., 2022). Second, we performed multiple sensitivity analyses to explore the uncertainty in the ICER. We found that the ICER remained similar when we used the maximal total cost and the median total cost instead of the mean total cost, suggesting that our conclusion that cranial implants are more cost-effective than autologous bone graft is robust when adjusting for these uncertainties in the ICER (Sanders et al., 2016; Jain et al., 2011). Third, the follow-up in our study was fairly long. In cost-effectiveness analyses, the time horizon should ideally cover the full period in which the intervention may affect either the costs or the effectiveness, as a restricted follow-up period may lead to an unfavorable representation of the ICER (Kim et al., 2017). Our study also has limitations. First, the use of DBC codes to collect cost data can be a limitation. DBC codes are packages of healthcare products required to formulate a diagnosis and provide treatment that hospitals can declare to the health care insurer (Struijs and Baan, 2011). However, as not every imaging scan or laboratory test is paid separately, the utilized healthcare products may sometimes differ from the declared healthcare products, leading to an over- or underestimation of the costs used in our analysis. Still, we believe that we would have reached the same conclusion regardless of a slight over- or underestimation of the costs used in our analysis. Second, the size of the autologous bone grafts group was larger than that of the cranial implants group, which could have resulted in an imbalance in baseline characteristics between the two groups. This may have affected the difference in revision surgery rate between the two groups and therefore our ICER calculations. To limit the effect of the group size difference, matched pair analysis should be considered in future developments of our study. Third, each cranioplasty implant material has its own advantages and disadvantages which may have affected our ICER calculations. For example, porous hydroxyapatite has higher risk of fractures but has a better profile in terms of infection control because it allows for biointegration, whereas there is OCEBM Level 3 Evidence that molding polymethyl methacrylate (PMMA) yields a much higher risk of infection and poorer cosmetic outcome than implanting custom made plates (Fricia et al., 2019; Ganau et al., 2020). Hence, health economic analyses conducted on international registries, such as the one from the United Kingdom (UK) and Ireland or Germany, should take into account those patient-specific and cranioplasty-specific aspects (Fountain et al., 2021; Sauvigny et al., 2021). Fourth, the generalizability of our results to countries using other currencies or other healthcare systems is limited. To draw more generalizable conclusions on the cost-effectiveness of cranial implants compared with autologous bone flaps, international studies with larger sample sizes are warranted.

## 5 Conclusion

The present study found that the use of cranial implants is at a lower cost and more clinically effective than the use of autologous bone flaps. Although cranial implants themselves are not significantly cheaper than autologous bone grafts, in the long-term they are cheaper and more cost-effective since they have a lower revision surgery rate and therefore lower total health care costs.

## Funding

This research did not receive any specific grant from funding agencies in the public, commercial, or not-for-profit sectors.

## Conflict of interest

The authors have no competing interests to declare.
